# Mechanistic study on components of *Angelica Pubescens-Taxillus Chinensis* in improving compression-induced intervertebral disc degeneration via piezo1/NF-κB signaling axis

**DOI:** 10.3389/fcell.2026.1935597

**Published:** 2026-10-01

**Authors:** Fei Yang, Chaoqun Feng, Guangye Li, Junwen Deng, Yunchuan Gou, Bingyi Yang, Ying Zhang, Xin Zhou, Chuan Leng, Maoyin Luo, Rigao Chen, Xiaohong Fan

**Affiliations:** 1 Department of Orthopedics, Hospital of Chengdu University of Traditional Chinese Medicine, Chengdu, China; 2 The Second Clinical Medical College, Guangzhou University of Chinese Medicine, Guangzhou, China; 3 Clinical Laboratory, Chongqing Yubei Hospital of Traditional Chinese Medicine, Chongqing, China

**Keywords:** angelica pubescens-taxillus chinensis, component-based Chinese medicine, NF-κB, nucleus pulposus, Piezo1, senescence

## Abstract

**Background:**

Abnormal mechanical load is a critical risk factor for intervertebral disc degeneration (IVDD), and nucleus pulposus (NP) cell senescence is a core pathogenic mechanism in IVDD. Piezo1 is a mechanosensitive ion channel, whereas the NF-κB pathway promotes cellular senescence. The classical formula Duhuo Jisheng decoction has shown potential benefits in IVDD-related conditions, and its core herbal pair *Angelica Pubescens-Taxillus Chinensis* accounting for its pivotal therapeutic effects.

**Objective:**

This study aimed to identify the optimal concentration ratio of the *Angelica Pubescens-Taxillus Chinensis* component-based Chinese medicine (CBCM) for attenuating compression-induced NP cell senescence and elucidate its molecular mechanism.

**Methods:**

An *in vitro* NP cell senescence model was established using 1.0 MPa compression. Nine CBCM combinations were defined using an L9 (3^3^) orthogonal design. A CCK-8 assay was used to screen the optimal and suboptimal combinations. Subsequently, the effect of CBCM on senescence was evaluated using senescence-associated β-galactosidase staining, Western blotting of senescence-related proteins, and qRT-PCR analysis of senescence-associated secretory phenotype factors. Additionally, the impact of CBCM on the Piezo1 and NF-κB pathways was analyzed through Western blotting, qRT-PCR, and immunofluorescence double staining. The underlying mechanism was confirmed by rescue experiments employing a Piezo1 agonist and antagonist, as well as p65 knockdown and overexpression. *In vivo*, a rat tail disc compression-induced IVDD model was established. The CBCM was administered intraperitoneally. IVDD was evaluated via X-ray, MRI, and histology. Molecular mechanisms underlying senescence, ECM degradation, and the Piezo1/NF-κB axis were analyzed by ELISA and Western blotting. Molecular docking was performed to further validate plausible interactions of CBN, OST, and QR with Piezo1.

**Results:**

Compression effectively induced NP cell senescence and activated Piezo1 expression and NF-κB signaling. The CBCM concentration ratio that optimally suppressed senescence markers was quercetin at 50 μM, osthole at 6.25 μM, and columbianadin at 12.5 μM. Mechanistically, the optimized CBCM inhibited Piezo1 expression, NF-κB activation, and p65 nuclear translocation. Both p65 knockdown and Piezo1 inhibition mimicked CBCM’s anti-senescent effects, whereas p65 overexpression or Piezo1 agonism reversed these protective effects. These *in vitro* findings were corroborated in a rat tail compression model of IVDD. Molecular docking offered computational evidence supporting the potential direct binding of CBN, OST, and QR with Piezo1 and proteins involved in the NF-κB signaling pathway.

**Conclusion:**

These findings suggest that the optimal *Angelica Pubescens-Taxillus Chinensis* CBCM attenuates the compression-induced senescence-associated phenotype of NP cells *in vitro* and in a rat tail model by modulating Piezo1-mediated NF-κB signaling.

## Graphical Abstract

**Figure:**
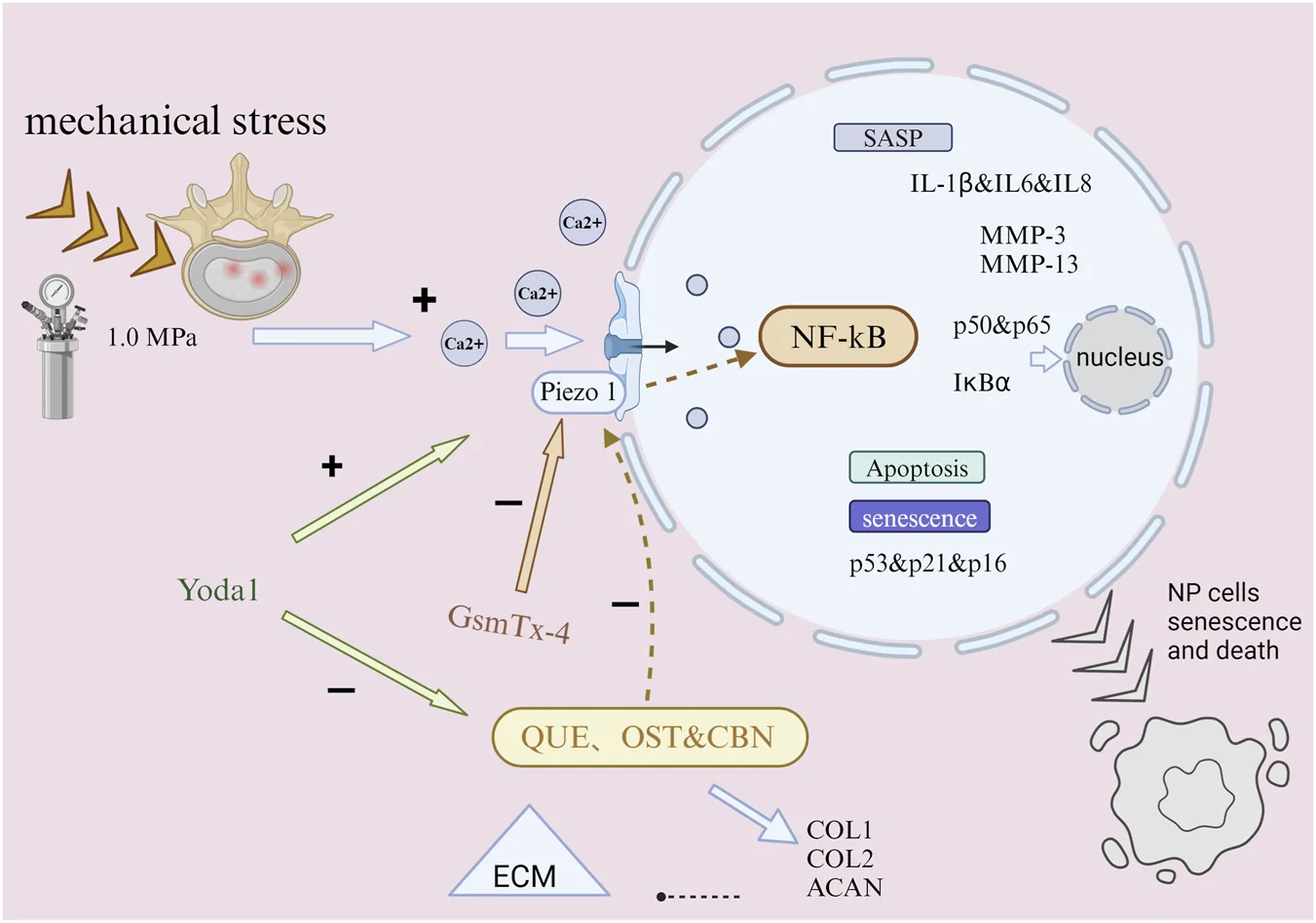

## 1 Introduction

Intervertebral disc degeneration (IVDD) is a chronic and multifactorial spinal disorder, considered the primary pathological basis of low back pain (LBP) (Vlaeyen et al., 2018; Binch et al., 2021; Knezevic et al., 2021). Globally, LBP affects approximately 80% of adults (Meucci et al., 2015; Knezevic et al., 2021), and the accelerated aging of the population further exacerbates its socioeconomic burden (Schofield et al., 2012; Hong et al., 2013). Weight-bearing and repetitive bending constitute established biomechanical risk factors for IVDD pathogenesis (Vergroesen et al., 2015). Within the disc microenvironment, the nucleus pulposus (NP) senses and responds to mechanical stimuli (Wu et al., 2022). Abnormal mechanical stress disturbs extracellular matrix (ECM) homeostasis in the NP, leading to NP cell senescence (Guerrero et al., 2021; Zhang et al., 2021; Sun et al., 2022; Wu et al., 2022; Xin et al., 2022). Cellular senescence is a stable cell-cycle arrest, characterized by enhanced senescence-associated β-galactosidase (SA-β-Gal) activity and the senescence-associated secretory phenotype (SASP). NP cell senescence is positively related to IVDD progression (Roberts et al., 2006; Zhang et al., 2020), and its targeted clearance alleviates IVDD progression in preclinical models (Novais et al., 2019; Huang et al., 2020). The mechanosensitive ion channel Piezo1, distributed in skeletal tissues including bone, articular cartilage, and intervertebral discs (Lee et al., 2014; Wang L. et al., 2020; Hendrickx et al., 2021), transduces mechanical signals into electrical or chemical signals (Zhao et al., 2018). Previous studies have shown that Piezo1 activation in NP cells acts as a trigger for senescence through complex molecular cascades, with the NF-κB pathway serving as a key downstream mediator of Piezo1-driven senescence signaling (Sun et al., 2020; Shi et al., 2022; Wu et al., 2022).

As higher-quality clinical studies emerge within TCM, therapeutic strategies targeting LBP have garnered increasing international attention (Zhang et al., 2017). *Duhuo Jisheng Decoction* (DHJSD), a classical TCM formula with documented efficacy against LBP (Zhang et al., 2016; Xiong et al., 2020), is recommended in the 2024 international guideline on TCM and integrative medicine using GRADE methodology for pain relief and functional improvement in LBP patients (Qin et al., 2024). However, its multi-component nature complicates mechanistic elucidation. A component-based Chinese medicine (CBCM) strategy offers a systematic approach. Active compounds from *Angelica pubescens* and *Taxillus chinensis*, a classical DHJSD herbal pair renowned for the therapeutic principle of “expelling wind-dampness, alleviating arthralgia, tonifying the liver and kidney, and replenishing qi and blood,” are widely utilized in clinical practice according to the “Qu-Zhi-Bu-Yi” doctrine. While modern studies have demonstrated that quercetin (QR) from *T. chinensis* and osthole (OST) and columbianadin (CBN) from *A. pubescens* inhibit inflammation and exert chondroprotective effects (Shao et al., 2021; Chen et al., 2023; Zhao et al., 2023), it remains unknown whether these components modulate the Piezo1/NF-κB signaling axis in NP cell senescence.

Therefore, this study aimed to identify the optimal concentration ratio of CBN, OST, and QR for anti-senescence efficacy, and to validate its therapeutic potential and underlying mechanism both *in vitro* and in a rat model of compression-induced IVDD.

## 2 Materials and methods

### 2.1 Cell culture and treatment

The immortalized human NP cell line was purchased from iCell Bioscience Inc., China. According to the cell authentication report, the origin, purity, and characteristics of the cells were confirmed. Specifically, the cells were derived from human intervertebral disc NP tissue, exhibited an elongated spindle-like morphology, and showed positive immunofluorescence staining for type II collagen, with a purity exceeding 90%. In addition, the report verified that the cells were free of contamination by HIV-1, HBV, HCV, *mycoplasma*, bacteria, yeast, and fungi. Cells were cultured in immortalized NP cell medium (iCell, China) and maintained at 37 °C in an incubator with 5% CO_2_. Senescence in NP cells was induced by 1.0 MPa pressure in the same culture condition, as described in previous studies (Wang K. et al., 2017; Li et al., 2018; Wu et al., 2019; Kang et al., 2020; Chi et al., 2023).

Based on literature review and preliminary experiments, QR was tested at concentrations of 12.5 μM, 25 μM, and 50 μM (Wang D. et al., 2020; Shao et al., 2021), OST at 3.125 μM, 6.25 μM, and 12.5 μM, and CBN at 12.5 μM, 25 μM, and 50 μM (Yang Y. et al., 2019; Chen et al., 2023; Feng et al., 2026). Based on orthogonal design methodology, different concentrations of active components from *Angelica Pubescens*-*Taxillus Chinensis* were matched and grouped. CBN, OST, and QR were purchased from MedChemExpress LLC, United States of America. An L9 (3^3^) orthogonal design was employed, and the complete grouping scheme with the exact concentrations of QR, OST, and CBN in all nine groups is provided in Table 1.

**TABLE 1:** Orthogonal design of quercetin (QR), osthole (OST), and columbianadin (CBN).

Group	QR (μM)	OST (μM)	CBN (μM)
1	12.5	3.125	12.5
2	12.5	6.25	25
3	12.5	12.5	50
4	25	3.125	25
5	25	6.25	50
6 (QZBY2)	25	12.5	12.5
7	50	3.125	50
8 (QZBY1)	50	6.25	12.5
9	50	12.5	25

### 2.2 Animal model establishment and treatment

After 1 week of acclimatization, a total of 60 male Sprague-Dawley rats were randomly divided into six groups: control, sham, model, low-dose, high-dose, and positive control, with ten rats in each group (n = 10). Three-month-old male Sprague-Dawley rats weighing 300–350 g were used. The control group received no modeling procedure, the sham group underwent skin puncture only, and the remaining groups all received the modeling intervention. The modeling procedure was performed under isoflurane inhalation anesthesia, using a rat-tail intervertebral disc bending-compression device (Haopai, China) (Xia et al., 2018; Ji et al., 2021) (Figure 5A). Kirschner wires were fixed percutaneously at the centers of the Co8 and Co10 vertebrae, positioned at a 40° inter-wire angle on the concave side of the curved tail, while a tension spring was mounted on the convex side. A polymer fixation plate was applied to prevent rotational movement of the tail and to minimize disruption to surrounding anatomical structures. Penicillin sodium was administered for the first 3 postoperative days. The compression apparatus was maintained for the 2-week modeling period and then was removed.

The components were dissolved in a vehicle consisting of 10% DMSO, 40% PEG300, 5% Tween-80, and 45% normal saline. Based on previous studies, QR at 50 mg·kg^-1^ alleviates IVDD progression in rats, while 100 mg·kg^-1^ shows enhanced efficacy without marked toxicity (Shao et al., 2021; Zhao et al., 2023). Similarly, OST and CBN were used at doses previously reported *in vivo* (Lim et al., 2014; Jayakumar et al., 2021; Chen et al., 2023; Jiang et al., 2023). The control, sham, and model groups received the vehicle alone. The low-dose group received a combined administration of QR 50 mg·kg^-1^, OST 20 mg·kg^-1^, and CBN 20 mg·kg^-1^ every other day, and the high-dose group was administered QR 100 mg·kg^-1^, OST 40 mg·kg^-1^, and CBN 40 mg·kg^-1^ on the same schedule. The positive control group was treated with GsmTx-4 at 270 μg·kg^-1^, administered once weekly. All treatments were given intraperitoneally for 8 weeks.

### 2.3 Cell viability

Cell viability was assessed using the CCK-8 assay kit (Biosharp, China). Cells were exposed to 1.0 MPa pressure for 12, 24, 36, or 48 h. The control and compression-treated cells were seeded into 96-well culture plates. The CCK-8 solution was added to each well. After further incubation for 2 h, the absorbance at 450 nm was measured using a microplate reader.

### 2.4 SA-β-gal staining

SA-β-gal staining was performed according to the manufacturer’s protocol (Solarbio, China). Briefly, cells were fixed with β-galactosidase staining fixative at room temperature for 15 min. After fixation, cells were washed three times with phosphate-buffered saline (PBS). Cells were then incubated overnight at 37 °C with prepared SA-β-gal staining working solution. Following incubation, cells were rinsed with PBS and imaged under an optical microscope. The stained cells were quantified using Image Pro software.

### 2.5 Immunofluorescence double staining of Piezo1 and p65

NP cells grown on coverslips were fixed and permeabilized. The cells were incubated overnight at 4 °C with a primary antibody against Piezo1 (1:400, Bioss, China). Following three washes with PBS, cells were incubated with a secondary antibody (1:200, Servicebio, China) for 50 min at room temperature. TSA signal amplification was then performed using FITC-Tyramide (1:500, Servicebio, China) for 10 min. Subsequently, cells were incubated overnight at 4 °C with an anti-p65 primary antibody (1:200, Bioss, China), followed by a secondary antibody (1:200, Servicebio, China) for 50 min. Nuclear counterstaining was conducted with DAPI (Servicebio, China) for 10 min. Images were acquired using a slide scanner (Olympus, Japan). Initial image browsing and qualitative analysis were performed using OlyVIA software (Olympus, Japan). Quantitative analysis was conducted using the Halo Image Analysis Platform (Indica Labs, USA).

### 2.6 Western blotting

NP cell samples were homogenized in pre-cooled RIPA lysis buffer. The supernatants were collected, and protein concentrations were determined using a BCA assay kit (Beyotime, China). Nuclear and cytoplasmic proteins were extracted using a nuclear and cytoplasmic protein extraction kit (Beyotime, China) according to the manufacturer’s instructions. Proteins were separated by SDS-PAGE and transferred to PVDF membranes. After blocking, the membranes were incubated overnight at 4 °C with primary antibodies against p16, p21, p53, Piezo1, aggrecan (ACAN), collagen I (COL1), collagen II (COL2), and β-actin. Following extensive washes, the membranes were incubated with a fluorescently labeled secondary antibody (Affinity, USA) for 2 h at room temperature. Finally, protein bands were visualized using a chemiluminescence imaging system and quantitatively analyzed with Gel-Pro Analyzer 4.0 software (Media Cybernetics, USA). The manufacturer, catalog number, host species, and working dilutions of the antibodies used for Western blotting are listed in Table 2 and 3.

**TABLE 2:** Antibodies used for *in vitro* Western blotting.

Target	Manufacturer	Catalog no.	Dilution	Host
p65	ABclonal, China	A19653	1:1000	Rabbit
p-p65	ABclonal, China	AP0475	1:1000	Rabbit
p16	ABclonal, China	A11651	1:2000	Rabbit
p21	ABclonal, China	A1483	1:1000	Rabbit
p53	ABclonal, China	A0263	1:2000	Rabbit
Piezo1	ABclonal, China	A23380	1:1000	Rabbit

**TABLE 3:** Antibodies used for *in vivo* Western blotting.

Target	Manufacturer	Catalog no.	Dilution	Host
p16	Bioss, China	Bs-23797 R	1:2000	Rabbit
p21	Proteintech, United States	28248-1-AP	1:1000	Rabbit
p53	Proteintech, United States	60283-2-Ig	1:2000	Rabbit
ACAN	Abcam, United Kingdom	ab313636	1:1000	Rabbit
COL1	Proteintech, United States	66761-1-Ig	1:1000	Rabbit
COL2	Affinity, United States	AF0135	1:1000	Rabbit
MMP-3	HuaBio, China	ER1706-77	1:1000	Rabbit
MMP-13	Proteintech, United States	18165-1-AP	1:1000	Rabbit
IκBα	Zhengneng, China	R380682	1:1000	Rabbit
p65	Proteintech, United States	10745-1-AP	1:1000	Rabbit
Piezo1	HuaBio, China	HA601100	1:1000	Rabbit
p-p65	Zhengneng, China	310,229	1:1000	Rabbit

### 2.7 Plasmid transfection and short interfering (si)RNA transfection

P65 plasmid transfection was performed using the target plasmid and the corresponding empty vector control (GenePharma, China). P65 silencing was achieved via P65-targeting siRNA and the corresponding negative controls (GenePharma, China). All transfections were performed using Lipofectamine^TM^ 3,000 (Invitrogen, USA), according to the manufacturer’s protocol. The transfection and siRNA are provided in Supplementary Table S1.

### 2.8 Quantitative real-time PCR

Total RNA was extracted from frozen specimens using MolPure Cell/Tissue Total RNA Kit (Yeasen, China), and the process of RNA reverse transcription was performed with PrimeScript^TM^ RT Reagent Kit (Takara, Japan). The resulting cDNA was amplified by quantitative real-time PCR using TB Green Premix Ex Taq ^TM^ (TaKaRa Bio) on a QuantStudio^TM^ Real-Time PCR Detection System (ThermoFisher, USA). Relative mRNA expression levels were calculated using the 2^-△△CT^ method with GAPDH as the internal control. Primer sequences are listed in Table 4.

**TABLE 4:** Primer sequences used for qRT-PCR.

Gene symbol	Forward primer (5′–3′)	Reverse primer (5′–3′)	Size (bp)
GAPDH	TGA​CTT​CAA​CAG​CGA​CAC​CCA	CAC​CCT​GTT​GCT​GTA​GCC​AAA	121
IL-6	CAC​TGG​TCT​TTT​GGA​GTT​TGA​G	GGA​CTT​TTG​TAC​TCA​TCT​GCA​C	101
IL-8	AAC​TGA​GAG​TGA​TTG​AGA​GTG​G	ATG​AAT​TCT​CAG​CCC​TCT​TCA​A	147
MMP-3	TTC​CTG​GCA​TCC​CGA​AGT​GG	AGC​CTG​GAG​AAT​GTG​AGT​GGA​G	148
MMP-13	CAC​TTT​ATG​CTT​CCT​GAT​GAC​G	TCT​GGC​GTT​TTT​GGA​TGT​TTA​G	95
p65	GGG​AAT​CCA​GTG​TGT​GAA​GAA​GC	GCA​CAG​CAT​TCA​GGT​CGT​AGT​C	125
Piezo1	TCT​GTG​GTC​ATC​CCT​AAT​CTC​TTC​C	ATA​CGC​ACG​CCG​AGG​TAG​TC	116

GAPDH, was used as the internal control.

### 2.9 X-ray

X-ray examinations were performed in each group at the end of the modeling period to evaluate whether the modeling was successful. Anesthesia was induced by intraperitoneal injection of 2% sodium pentobarbital, after which the tail was positioned within the detection field. The disc height index (DHI%) was measured from all available radiographs.

### 2.10 MRI

Three rats per group were randomly selected for MRI examination 24 h after the final administration. T2-weighted sagittal MRI of the rat tail was performed using a 1.5-T MRI scanner with a dedicated rodent coil (TR: 3,800 ms, TE: 107.1 ms, TI: 0, FOV: 320 × 340 mm, ST: 0.6 mm). IVDD was then graded according to the Pfirrmann classification system (Pfirrmann et al., 2001).

### 2.11 Histological processing

Following imaging, rats were euthanized, and the compressed tail disc segments along with adjacent vertebrae were harvested. The collected tissue samples from each group were fixed, then sequentially decalcified, dehydrated, cleared, and paraffin-embedded. Consecutive 5-μm sections were prepared for histological analysis. For general morphological evaluation, sections were stained with Hematoxylin and Eosin (HE). To assess proteoglycan content, adjacent sections were stained with Safranin O-Fast Green. All stained sections were examined under a light microscope. Histological degeneration was scored using ORS grading scheme (Lai et al., 2021).

### 2.12 ELISA

NP tissue was homogenized, and the levels of SA-β-gal activity and SASP factors were measured by ELISA according to the manufacturer’s instructions (Zhuocai, China).

### 2.13 Molecular docking

The human Piezo1 cryo-EM structure (PDB ID: 8YEZ) was obtained from the Protein Data Bank. Prepared three-dimensional structures of CBN, OST, and QR were docked under identical receptor and search settings using AutoDock Vina v1.2.5 (Trott and Olson, 2010; Eberhardt et al., 2021). Hydrogen bonds, hydrophobic contacts, and π–π interactions were identified using the Protein–Ligand Interaction Profiler (PLIP) v3.0.1 (Adasme et al., 2021), and structures were visualized using PyMOL.

### 2.14 Statistical analysis

Statistical analysis was performed using SPSS statistical software (version 25.0). Data are presented as mean ± SEM. All radiographic and MRI evaluations were performed independently by two observers blinded to group allocation, with any discrepancies adjudicated by a senior investigator. Comparisons among multiple groups were analyzed by One-Way ANOVA, followed by the LSD test for homogeneous variances or Tamhane’s T2 test for heterogeneous variances. Differences with *P < 0.05* were considered statistically significant.

## 3 Results

### 3.1 Effects of CBCM on compression-induced cytotoxicity in human NP cells

Compression was used to establish the NP cell senescence model *in vitro*. The CCK-8 assay demonstrated that CBCM exhibited no cytotoxicity on NP cells at tested concentrations under normal pressure, except for Group 9, which significantly inhibited proliferation at 24, 36 and 48 h. Under compression conditions, NP cells treated with CBCM for 36 h maintained good viability, whereas a universal decline in NP cells activity occurred after 48 h (Figure 1A). Therefore, the 1.0 MPa compression for 36 h was consequently established as the modeling condition. Screening identified Group 8 as the optimal ratio, providing the strongest protection against compression-induced viability loss, and Group 6 as the suboptimal ratio; both ratios were subsequently confirmed by anti-senescence readouts (Figures 1B–D). These two ratios were used in subsequent experiments and are hereinafter referred to as QZBY1 and QZBY2 (Table 1).

**FIGURE 1:**
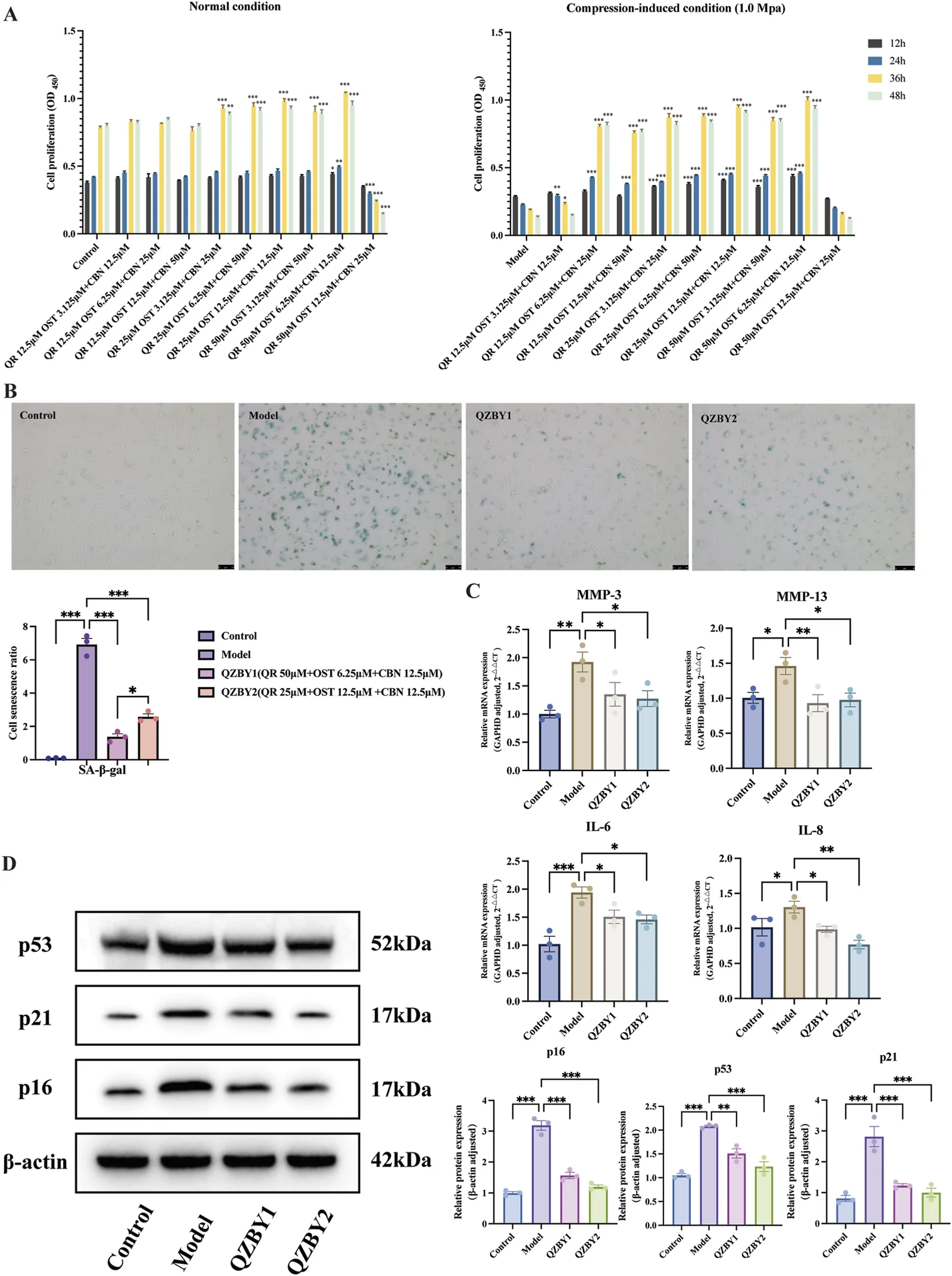
Effects of CBCM on NP cell viability and senescence. Note: **(A)** Proliferation of NP cells treated with various CBCM ratios under normal or compression conditions was measured by CCK-8 assay at 12, 24, 36, and 48 h. **(B)** Representative images (75 μm) and quantitative analysis of SA-β-gal staining. **(C)** Relative mRNA expression levels of SASP factors MMP-3, MMP-13, IL-6, and IL-8. **(D)** Relative protein expression levels of cell cycle-related proteins p53, p21, and p16. *P < 0.05, **P < 0.01, ***P < 0.001. QZBY1 (Group 8) contained QR 50 μM, OST 6.25 μM, and CBN 12.5 μM; QZBY2 (Group 6) contained QR 25 μM, OST 12.5 μM, CBN 12.5 μM.

### 3.2 Effects of CBCM on compression-induced senescence in human NP cells

Next, we explored the anti-senescence effects of CBCM. SA-β-gal staining revealed that compression significantly increased the proportion of senescent cells in the model group. Both QZBY1 and QZBY2 ratios attenuated this phenotype, with QZBY1 demonstrating superior efficacy (Figure 1B). QRT-PCR analysis showed that the mRNA levels of SASP factors, namely IL-6, IL-8, MMP-3, and MMP-13, were markedly increased in human NP cells following compression exposure. This compression-induced upregulation in SASP expression was suppressed by both QZBY1 and QZBY2 (Figure 1C). Western blot analysis indicated that compression upregulated p53, p21, and p16, whereas QZBY1 and QZBY2 downregulated all three proteins (Figure 1D). Taken together, these findings indicate that CBCM attenuates the compression-induced senescence-associated phenotype in human NP cells.

### 3.3 Effects of CBCM on compression-induced Piezo1 expression and NF-κB signaling activation in human NP cells

We first examined Piezo1 expression and NF-κB signaling activation in human NP cells under compression. As shown in Figure 2A, compared with the control group, the model group exhibited significantly elevated mRNA expression of p65 and Piezo1 in NP cells. Both QZBY1 and QZBY2 groups substantially decreased p65 and Piezo1 mRNA expression. Western blotting revealed markedly increased Piezo1 protein expression in model group, which was significantly reversed by QZBY1 and QZBY2 (Figure 2B). In addition, Western blot analysis of nuclear and cytoplasmic fractions demonstrated that nuclear and cytoplasmic p65 and phospho-p65 (p-p65) expressions was significantly upregulated in the model group. QZBY1 and QZBY2 decreased nuclear and cytoplasmic p65 and p-p65 expressions (Figures 2C–F). Immunofluorescence detected upregulation of Piezo1 and nuclear translocation of NF-κB in the compression-induced model group. However, a marked decrease in the fluorescence intensity of Piezo1 and NF-κB was observed following QZBY1 or QZBY2 treatment (Figures 2G–J).

**FIGURE 2:**
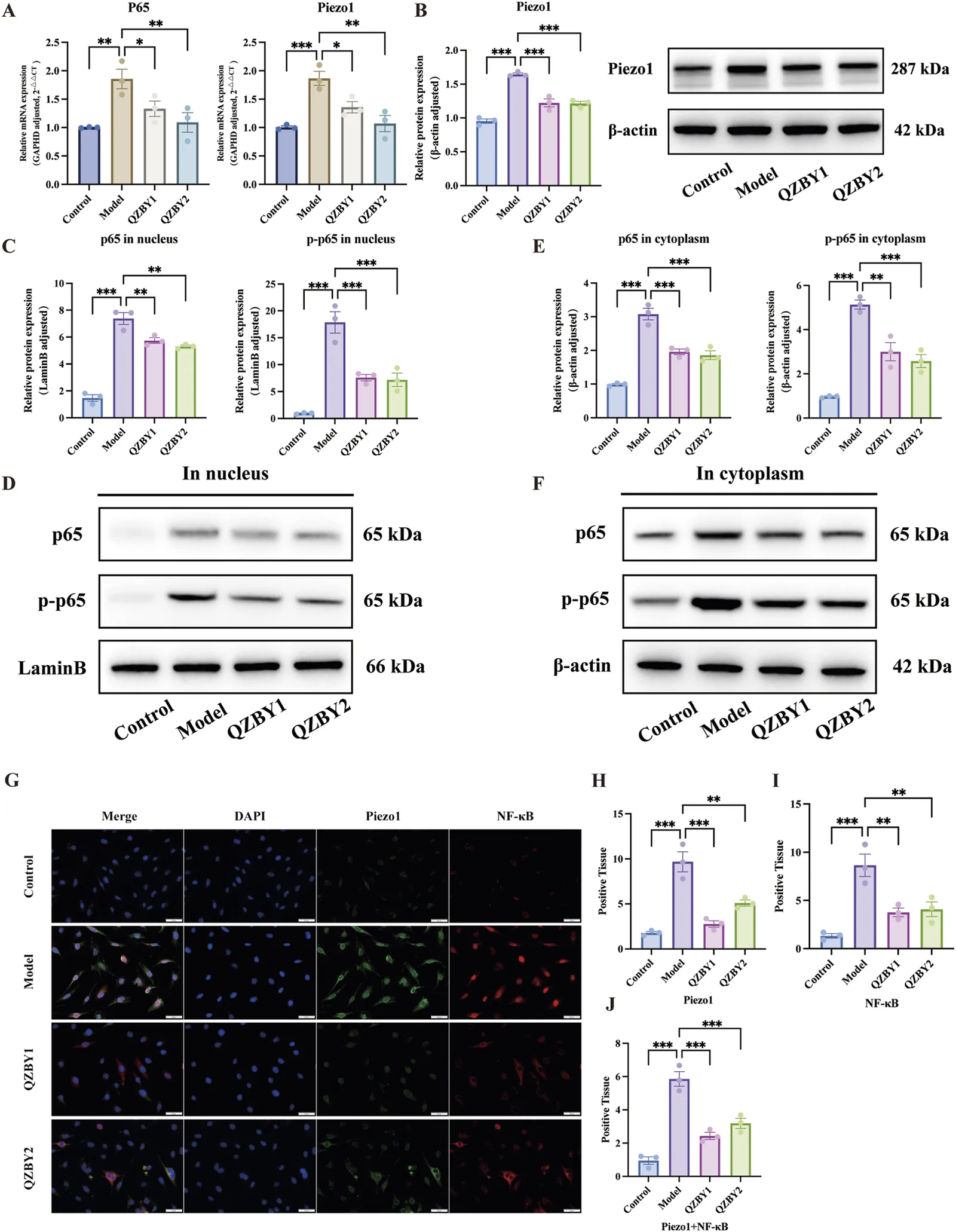
CBCM modulates Piezo1 and NF-κB signaling in compressed NP cells. Note: **(A)** Relative mRNA expression levels of p65 and Piezo1. **(B)** Relative protein expression level of Piezo1. **(C,D)** Relative protein expression levels of nuclear p65 and p-p65. **(E,F)** Relative protein expression levels of cytoplasmic p65 and p-p65. **(G)** Representative immunofluorescence double staining images for Piezo1 (green) and NF-κB signaling (red) (50 μm). **(H–J)** Quantitative analysis of Piezo1 fluorescence intensity and the nuclear-to-cytoplasmic ratio of p65 (NF-κB). *P < 0.05, **P < 0.01, ***P < 0.001.

### 3.4 Role of NF-κB signaling in CBCM-mediated anti-senescence in compression-exposed human NP cells

The above results indicated that CBCM alleviated compression-induced senescence in human NP cells. Based on these findings, we proposed that the ameliorative effect of CBCM is mediated through the NF-κB signaling pathway. To further investigate this mechanism, we interfered with NF-κB signaling by knocking down p65 using siRNA and overexpressing p65 via plasmid transfection, respectively. Transfection efficiency was validated by qRT-PCR, which showed that siRNA-p65 significantly decreased p65 mRNA expression, while pcDNA-p65 increased it (Figure 3E). SA-β-gal staining confirmed that siRNA-p65 reduced the proportion of senescent cells, and pcDNA-p65 increased cellular senescence even in the presence of QZBY1 treatment (Figures 3A,B). Furthermore, qRT-PCR analysis of SASP factors revealed that siRNA-p65 downregulated the mRNA levels of MMP-3, MMP-13, IL-6, and IL-8 compared with the siRNA-NC group, and pcDNA-p65 reversed the protective effects of QZBY1 (Figure 3C). Western blot analysis showed that transfection with siRNA-p65 significantly decreased the protein levels of p53, p21 and p16, while pcDNA-p65 upregulated all three proteins (Figures 3D,F). Meanwhile, p65 silencing markedly inhibited both nuclear and cytoplasmic protein levels of p65 and p-p65, while pcDNA-p65 increased nuclear and cytoplasmic p65 and p-p65 levels even in QZBY1-treated cells (Figures 3G,H). Together, these genetic manipulations confirm that NF-κB signaling plays a critical role in mediating the anti-senescence effects of CBCM.

**FIGURE 3:**
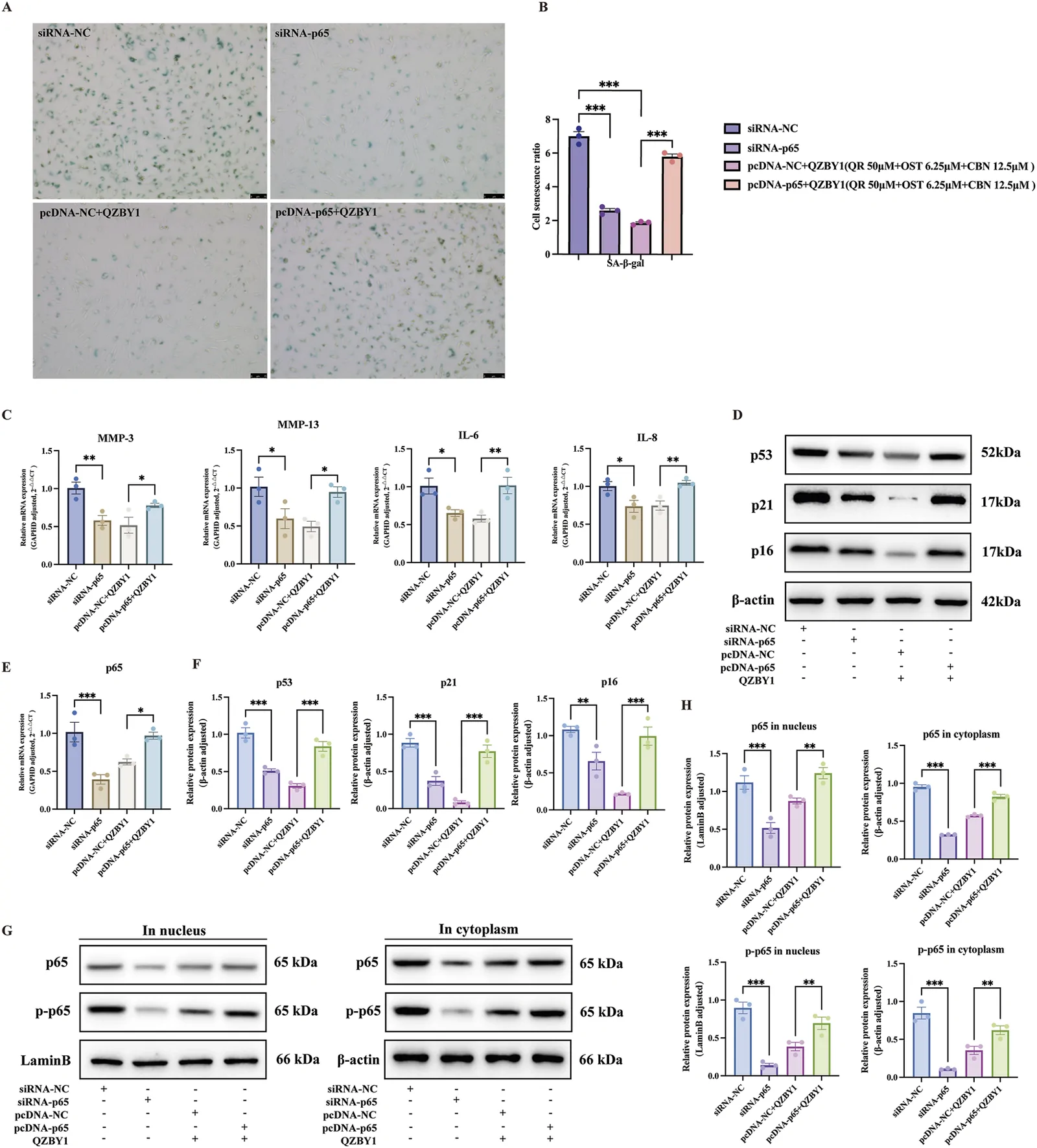
CBCM alleviates compression-induced senescence in NP cells by modulating NF-κB signaling. Note: **(A,B)** Representative images and quantitative analysis of SA-β-gal staining (75 μm). **(C)** Relative mRNA expression levels of SASP factors MMP-3, MMP-13, IL-6, and IL-8. **(D,F)** Relative protein expression levels of senescence markers p53, p21, and p16. **(E)** Relative mRNA expression level of p65. **(G,H)** Relative protein expression levels of nuclear and cytoplasmic p65 and p-p65. *P < 0.05, **P < 0.01, ***P < 0.001.

### 3.5 Role of Piezo1-mediated NF-κB signaling in ameliorating compression-induced senescence in human NP cells

To investigate whether Piezo1-mediated NF-κB signaling drives CBCM’s protective effects on human NP cells under compression, we targeted this axis using a Piezo1 inhibitor and agonist. When the Piezo1 inhibitor GsmTx-4 or the agonist Yoda1 was used on NP cells, the cells were pre-incubated for 2 h before compression exposure. SA-β-gal staining showed that both GsmTx-4 and QZBY1 significantly reduced the proportion of senescent cells compared with the model group, while Yoda1 co-treatment reversed the protective effect of QZBY1 (Figures 4A,B). Immunofluorescence analysis confirmed that the compression-induced upregulation of Piezo1 and the nuclear translocation of p65 were suppressed by GsmTx-4 and QZBY1. In contrast, Yoda1 not only reversed the inhibitory effect of QZBY1 but also promoted NF-κB nuclear translocation (Figures 4C–F). At the transcriptional level, qRT-PCR analysis revealed that GsmTx-4 downregulated the expression of MMP-3, MMP-13, IL-6, IL-8, and p65, while QZBY1 more potently suppressed all these SASP factors. In contrast, Yoda1 counteracted the effects of QZBY1 by upregulating these genes (Figure 4G). Among the senescence-related proteins, the protein levels of p53, p21, and p16 were increased following compression treatment. However, this increase was attenuated by GsmTx-4 intervention, with QZBY1 exhibiting even stronger suppression of all senescence-related protein markers. Again, Yoda1 reversed the QZBY1-mediated downregulation of these proteins (Figure 4H). Subcellular fractionation analysis demonstrated that both GsmTx-4 and QZBY1 reduced the levels of nuclear and cytoplasmic p65 and p-p65, whereas Yoda1 increased them (Figures 4I,J). Collectively, these results indicate that Piezo1 activation mediates compression-induced senescence via NF-κB signaling, and CBCM can interrupt this pathological process by modulating the Piezo1/NF-κB axis.

**FIGURE 4:**
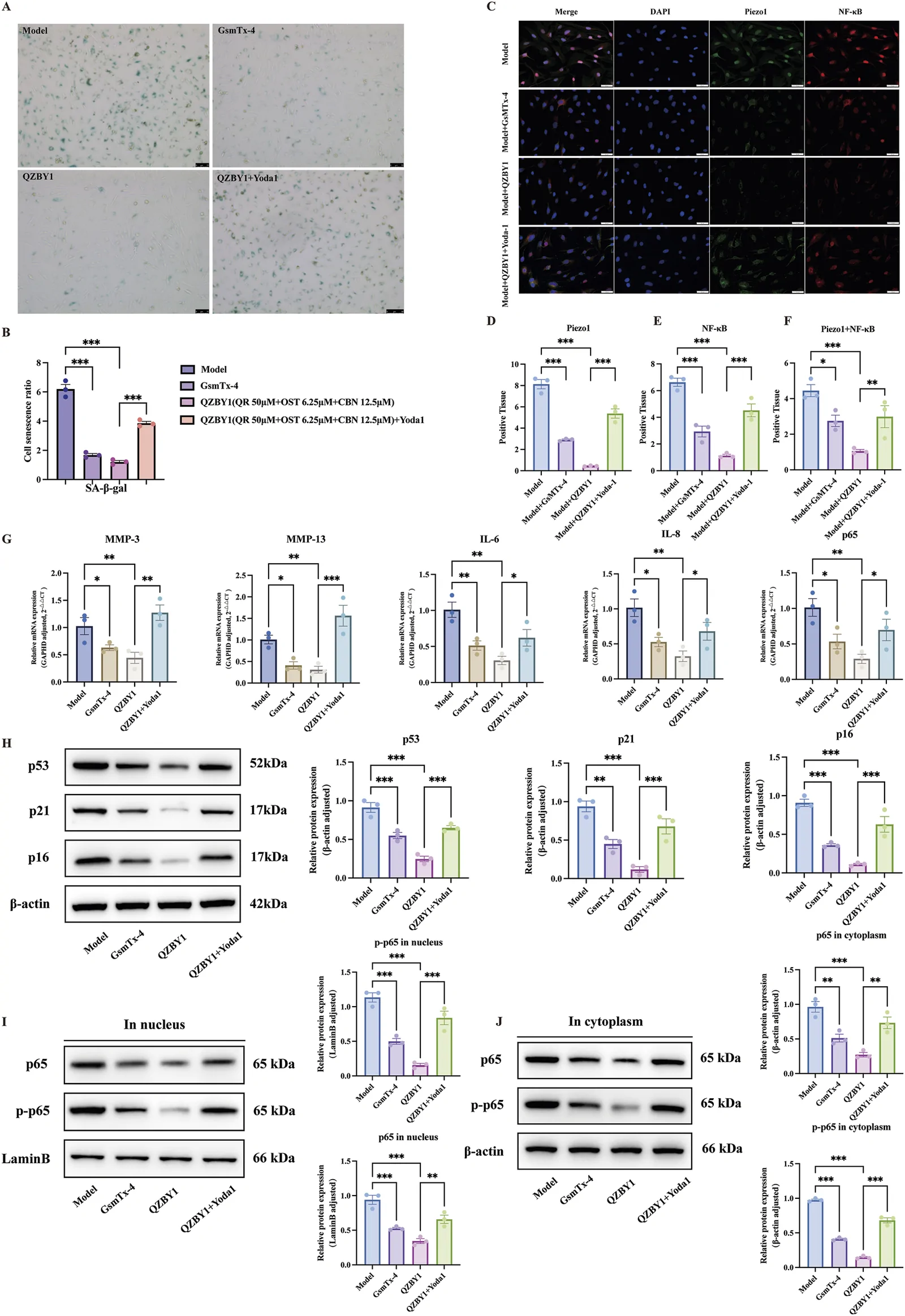
CBCM attenuates NP cell senescence by suppressing Piezo1-mediated NF-κB signaling. Note: **(A,B)** Representative images and quantitative analysis of SA-β-gal staining (75 μm). **(C)** Representative immunofluorescence double staining images for Piezo1 (green) and NF-κB signaling (red) (50 μm). **(D–F)** Quantitative analysis of Piezo1 fluorescence intensity and the nuclear-to-cytoplasmic ratio of p65 (NF-κB). **(G)** Relative mRNA expression levels of SASP factors MMP-3, MMP-13, IL-6, IL-8, and p65. **(H)** Relative protein expression levels of cell cycle-related proteins p53, p21, and p16. **(I,J)** Relative protein expression levels of nuclear and cytoplasmic p65 and p-p65. *P < 0.05, **P < 0.01, ***P < 0.001.

### 3.6 Effects of CBCM on compression-induced NP senescence and ECM degradation *in vivo*

Building on our *in vitro* findings, we next assessed the therapeutic efficacy of CBCM in a rat model of compression-induced IVDD. Throughout the treatment period, body weight increased steadily in all groups, with no significant between-group differences at any time point (Supplementary Figure S1). X-ray imaging showed the modeling using the tail disc bending-compression method (Figure 5A). On T2-weighted MRI, control and sham discs exhibited high signal intensity and clear morphology, whereas model discs showed markedly reduced NP signal, structural disorganization, and loss of disc height. Both low-dose (QZBY-L) and high-dose (QZBY-H) CBCM treatment preserved NP signal intensity and structure, with effects comparable to the positive control GsmTx-4, and partially restored the reduced disc height index (DHI%) (Figure 5B). Histologically, HE staining revealed that control and sham discs maintained a gel-like NP rich in vacuolated notochordal cells and a well-organized annulus fibrosus. In contrast, model discs displayed severe degeneration, characterized by fibrocartilaginous replacement of the NP, decreased cellularity, and loss of the border. CBCM treatment dose-dependently preserved NP cellularity and gel-like morphology (Figure 5C). Consistently, Safranin O-Fast Green staining demonstrated severe proteoglycan loss in model discs, which was markedly attenuated by CBCM treatment (Figure 5D). In addition, Pfirrmann grades and ORS histological scores are also improved, further confirming its protective effect on disc structure (Table 5; Figure 5D). At the molecular level, ELISA showed that compression-induced increases in SA-β-gal activity and SASP factors were significantly suppressed by both doses of CBCM (Figure 5E). Western blot analysis further revealed that CBCM downregulated the senescence-associated proteins p53, p21, and p16, which were upregulated in the model group (Figures 5F,G). CBCM also counteracted compression-induced ECM degradation. Compared with controls, model discs showed a pronounced reduction in ACAN and COL2 and both QZBY-L and QZBY-H, as well as the positive control, significantly restored their levels (Figures 5H,I). COL1 showed similar directional changes. Conversely, the expression of matrix-degrading enzymes MMP-3 and MMP-13 was markedly elevated in model discs. While QZBY-L significantly lowered MMP-3 but not MMP-13, QZBY-H and the positive control significantly suppressed both enzymes (Figures 5J,K).

**FIGURE 5:**
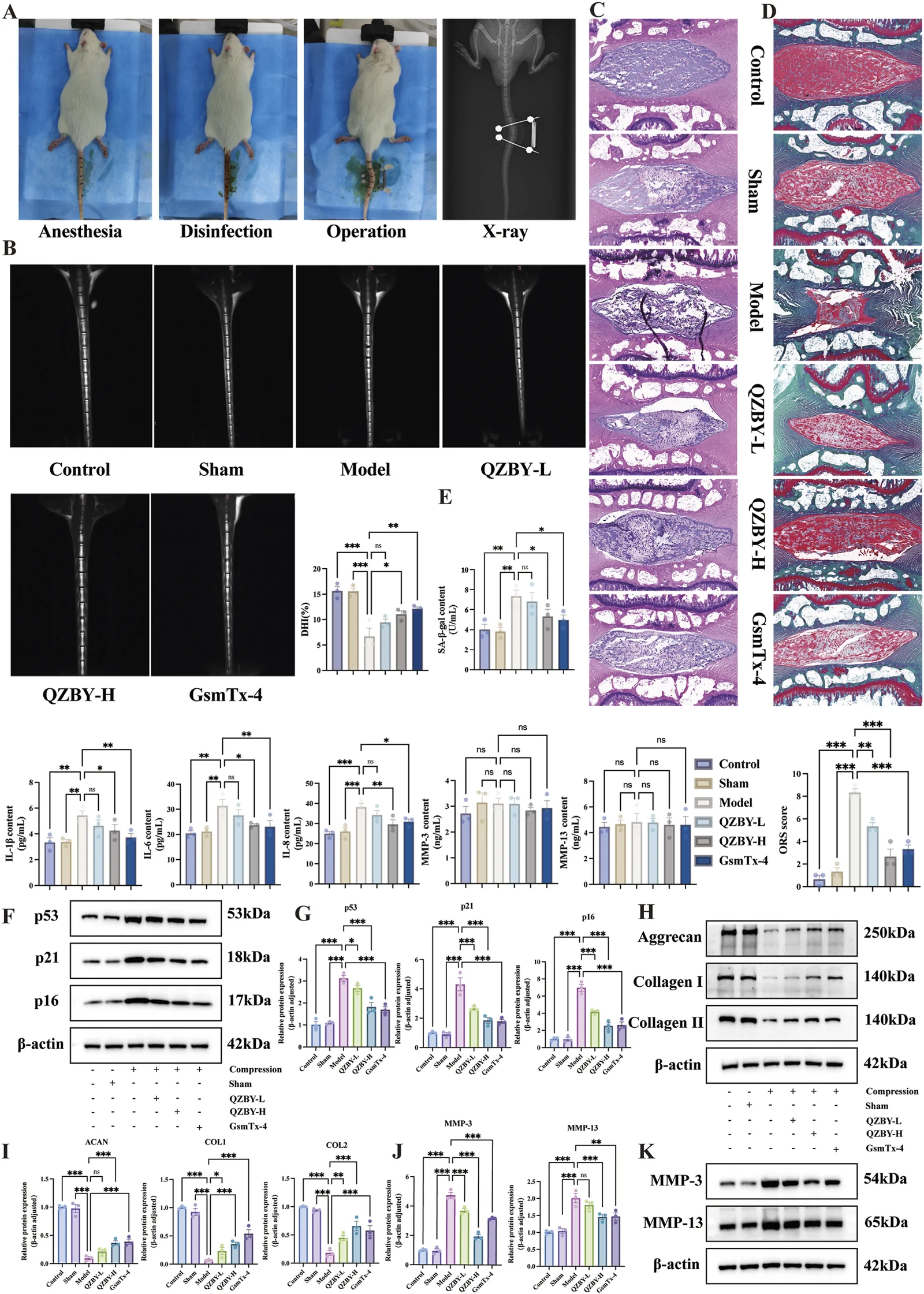
CBCM attenuates NP senescence and ECM degradation *in vivo*. Note: **(A)** Modeling procedure using the rat tail intervertebral disc bending-compression method. **(B)** Representative MRI scan and DHI% of the rat tail after treatment. **(C,D)** HE staining and Safranin O-Fast Green staining of the modeled intervertebral disc segment. **(E)** ELISA and ORS score. **(F,G)** Relative expression levels of senescence-associated proteins. **(H,I)** Relative expression levels of ECM-associated proteins. **(J,K)** Relative expression levels of MMP-3 and MMP-13 proteins. *P < 0.05, **P < 0.01, ***P < 0.001.

**TABLE 5:** Pfirrmann grading of rat caudal discs (*mean ± SEM*, n = 3).

Group	Dosage (mg·kg^-1^)	Pfirrmann grade
Control	0	1.33 ± 0.58^**^
Sham	0	1.67 ± 0.58^**^
Model	0	4.33 ± 0.58
QZBY-L	QR 50 + OST 20 + CBN 20	3.00 ± 0.00^**^
QZBY-H	QR 100 + OST 40 + CBN 40	2.00 ± 0.00^**^
GsmTx-4	0.27	2.33 ± 0.58^**^

**P < 0.01. For cell-based quantitative analyses, n denotes independent experiments. For *in vivo* analyses, n denotes individual animals.

### 3.7 Effects of CBCM on the NF-κB pathway through inhibition of Piezo1 *in vivo*

Western blot analysis demonstrated that compression modeling significantly increased Piezo1 expression in NP tissue relative to controls. This elevation was dose-dependently attenuated by CBCM treatment (Figures 6A,B). Concurrently, the NF-κB pathway was activated in model discs, as evidenced by increased p65 and p-p65 accompanied by decreased IκBα, consistent with enhanced IκBα degradation. Both QZBY-L and QZBY-H effectively counteracted this activation (Figures 6A,B).

**FIGURE 6:**
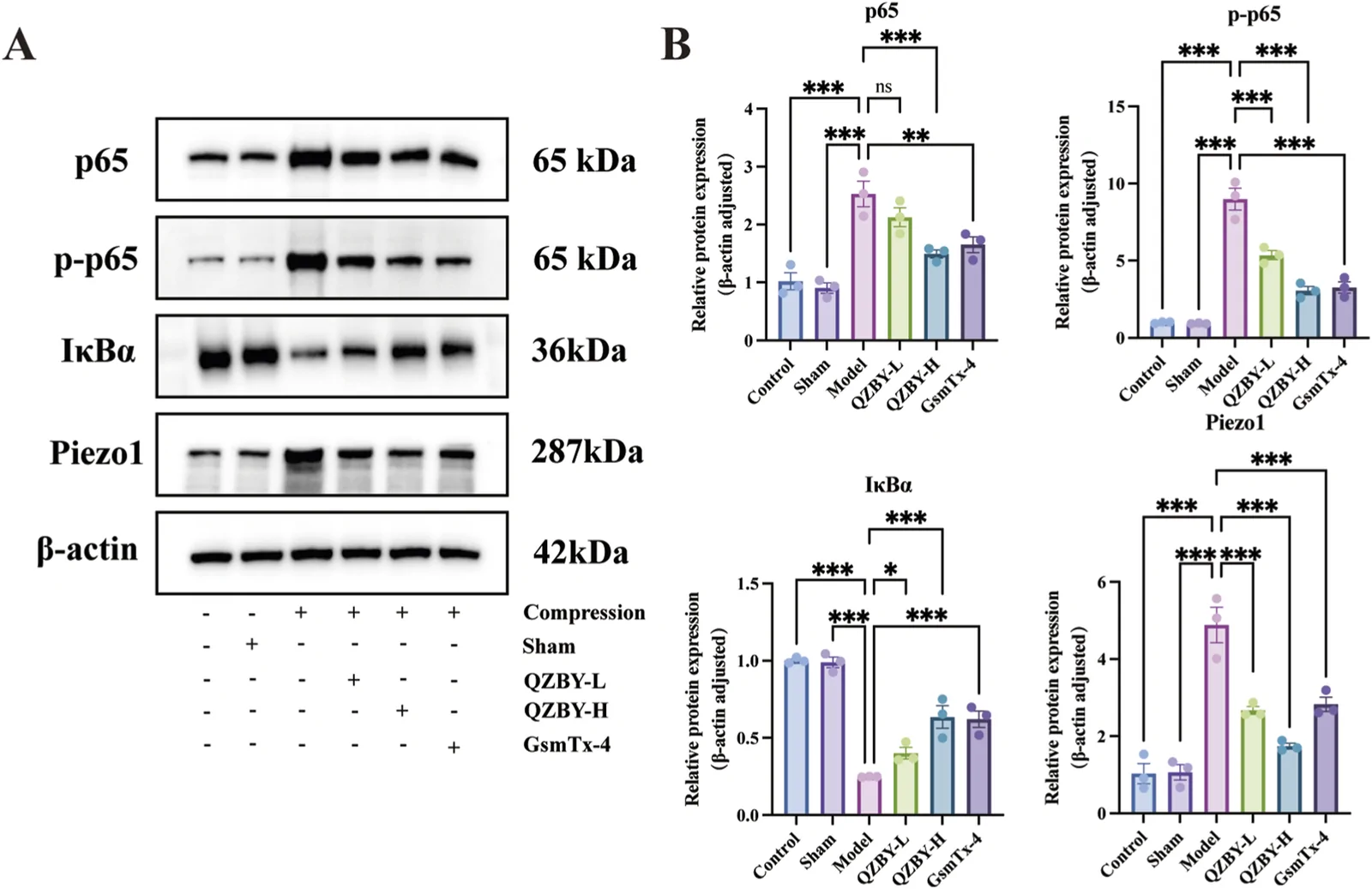
CBCM attenuates NP senescence by suppressing Piezo1 and NF-κB signaling *in vivo*. Note: **(A)** Representative western blot bands and relative protein expression levels of p65, p-p65, IκBα, and Piezo1 in NP tissue. **(B)** Statistical results of the relative expression levels of p65, p-p65, IκBα, and Piezo1 in NP tissue. *P < 0.05, **P < 0.01, ***P < 0.001. n = 10 rats per group.

### 3.8 Molecular docking suggests candidate interaction modes between CBCM components and Piezo1

Under identical docking conditions, CBN, QR, and OST yielded minimum Vina scores of −8.380, −7.699, and −7.390 kcal/mol, respectively (Figure 7; Supplementary Table S2). CBN occupied a hydrophobic pocket at the interface of adjacent Piezo1 subunits, with hydrophobic contacts involving Leu 2008, Phe 2012, and Ile2187 and a T-shaped π–π interaction with Phe 1972. OST showed two π–π interactions with Phe1972 and hydrophobic contacts with Phe2012 and Ile2184. QR formed a hydrogen bond with Leu2115, a π–π interaction with Phe 2012, and hydrophobic contacts involving Phe 1972, Phe 2012, Phe2114, Leu2115, Leu2118, Ile2184, and Ile2187. These docking results offered computational evidence supporting the potential direct binding of CBN, OST, and QR with Piezo1 and proteins involved in the NF-κB signaling pathway.

**FIGURE 7:**
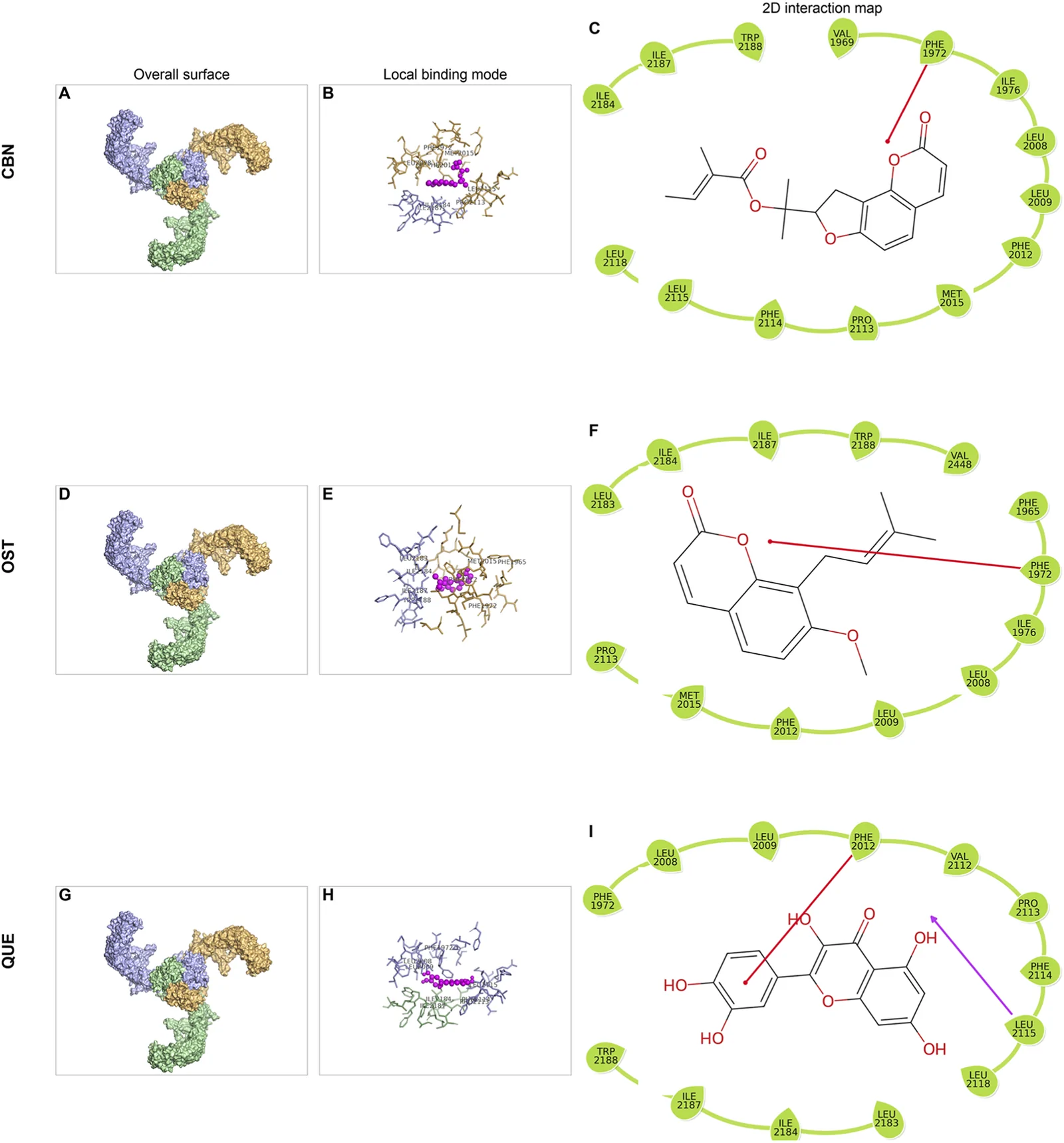
Molecular docking of CBN, OST, and QR with Piezo1. Note: **(A–C)** Overall surface, local binding mode, and 2D interaction map of CBN. **(D–F)** Overall surface, local binding mode, and 2D interaction map of OST. **(G–I)** Overall surface, local binding mode, and 2D interaction map of QR.

## 4 Discussion

This study identified an optimized combination of key bioactive components from the classical herbal pair *Angelica Pubescens* and *Taxillus Chinensis*, and elucidated its efficacy in alleviating compression-induced senescence in human NP cells via inhibition of the Piezo1/NF-κB signaling axis. The therapeutic efficacy and mechanistic insights gained *in vitro* were corroborated *in vivo*.

Abnormal mechanical loading is a central initiating factor in IVDD (Adams et al., 2000). NP tissue is highly sensitive to mechanical load. Pressure and ECM stiffness are the main mechanical cues of the microenvironment in the NP tissue (Bian et al., 2017). NP cells prefer a softer ECM, while a stiffer matrix leads to NP cell senescence (Fearing et al., 2019; Wang et al., 2021). NP cell senescence has thus become a central topic in IVDD research (Yang M. et al., 2019; Shi et al., 2022). In the present study, a compression intensity of 1.0 MPa applied for 36 h was employed to simulate pathological mechanical loading. According to previous literature, this regimen effectively induces not only cellular senescence and inflammatory activation in NP cells, but also mitochondrial dysfunction, oxidative stress, and apoptosis (Kang et al., 2020). These findings underscore the role of abnormal mechanical stress in driving multiple degenerative processes within the intervertebral disc microenvironment.

Piezo channels have provided critical insight into mechanotransduction mechanisms (Coste et al., 2010). First reported in 2010 and later recognized in the 2021 Nobel Prize, Piezo1 has been increasingly implicated in the pathophysiology of common musculoskeletal disorders (Lei et al., 2024). Accumulating evidence indicates an association between IVDD and the mechanosensitive ion channel Piezo1. Sun et al. reported that Piezo1 activation promotes NLRP3 inflammasome formation in NP cells through the Ca^2+^/NF-κB pathway, thereby exacerbating inflammatory responses (Sun et al., 2020). Under mechanical stress, Wu et al. demonstrated that Piezo1 initiates a self-amplifying loop of NF-κB and periostin, which accelerates NP cell senescence and contributes to IVDD progression (Wu et al., 2022). Furthermore, Piezo1 stimulates the expression of catabolic enzymes and facilitates ECM degradation in NP cells via NF-κB activation (Wang J. et al., 2017; 2019; Sun et al., 2020). Our results also demonstrate that compression significantly upregulates Piezo1 expression and activates the NF-κB signaling, inducing NP cell senescence.

IVDD is closely associated with various spinal disorders, including disc herniation, spinal stenosis, degenerative spondylolisthesis, and scoliosis, imposing a substantial socioeconomic burden (Hartvigsen et al., 2018). Currently, effective treatments specifically targeting the underlying mechanisms of IVDD remain under extensive investigation (Zhang et al., 2020; Yang et al., 2022). In this context, multi-target intervention offers a promising alternative to a single anti-senescence compound by better aligning with the complex and multifactorial pathophysiology of IVDD. Notably, CBCM exhibited stronger anti-senescence effects than the Piezo1 inhibitor GsmTx-4 *in vitro*. During the progression of IVDD, various modes of RCD exist within the intervertebral disc, such as apoptosis, pyroptosis, senescence, and autophagy-dependent cell death, which can occur independently or in combination (Yang et al., 2022). This study employed an orthogonal design methodology to determine the optimal ratio of QR, OST, and CBN. This approach transcends conventional strategies that focus on single compounds or crude extracts, providing a modernized and quantitative research framework that aligns with the holistic philosophy of multi-component and multi-target interventions in traditional Chinese medicine for disease prevention and treatment.

Notably, both genetic knockdown of p65 and pharmacological inhibition of Piezo1 reproduced the anti-senescence effects observed with CBCM treatment, while overexpression or activation of these molecules abolished its protective benefits. Moreover, inhibition of Piezo1 effectively suppressed NF-κB activation, indicating that Piezo1 acts upstream of the NF-κB pathway. These results indicate that CBCM attenuates the compression-induced senescence-associated phenotype, at least in part, through modulation of the Piezo1/NF-κB axis.

This study points toward valuable future research directions. While we delineated a central role for the Piezo1/NF-κB axis, the superior efficacy of CBCM compared with the Piezo1 inhibitor suggests its mechanism may extend beyond this pathway. The precise additional molecular targets and the integrative mechanism of action of the CBCM combination remain to be fully elucidated. Although we used an immortalized human NP cell line, immortalized cells may differ from primary NP cells in their senescence program and mechanosensitivity. Further validation in primary cells and clinical samples is required. In addition, genetic manipulation of Piezo1, Ca^2+^ influx measurements, IKKα/β phosphorylation analysis, and patch-clamp recordings are important for strengthening the mechanistic link from Piezo1 to NF-κB and for evaluating potential direct targeting. Nonetheless, this study transforms an empirical herbal pair into a traceable and optimizable therapeutic strategy by defining a precise component ratio and a core mechanistic axis, thereby offering a concrete paradigm for the modernized development of herbal medicines against complex degenerative diseases.

## 5 Conclusion

In summary, this study identified an optimized CBCM derived from the clinically effective herb pair *Angelica Pubescens-Taxillus Chinensis*, which alleviates compression-induced NP cell senescence through inhibition of the Piezo1/NF-κB signaling axis.

## Data Availability

The original contributions presented in the study are included in the article/Supplementary Material, further inquiries can be directed to the corresponding authors.
