# Supplementary material for: Mechanistic study on components of Angelica Pubescens-Taxillus Chinensis in improving compression-induced intervertebral disc degeneration via piezo1/NF-κB signaling axis
Source: Front Cell Dev Biol. 2026 Oct 1;14:1935597. doi: 10.3389/fcell.2026.1935597 (PMC13668827; doi:10.3389/fcell.2026.1935597)
Supplement: Supplementary file 1 [file DataSheet1.pdf]

Supplementary Table S1. siRNA sequences used for transfection.

Group	Catalog No.	Supplier	Sequence (5'→3')
**siRNA-p65**	RELA-Homo-1149	GenePharma	Sense:<br>GCACCAUCAACUAUGAUGATT<br>Antisense:<br>UCAUCAUAGUUGAUGGUGCTT

Supplementary Table S2. Molecular docking scores and PLIP-detected interactions of CBCM components with Piezo1.

Compound	Minimum Vina score (kcal/mol)	PLIP-detected interactions
**CBN**	−8.380	Hydrophobic: Leu2008 (3.23 Å), Phe2012 (3.60 Å), and Ile2187 (3.39 Å); T-shaped π–π: Phe1972 (4.72 Å); no PLIP-defined hydrogen bond.
**OST**	−7.390	π–π: Phe1972 (4.11 and 4.48 Å); hydrophobic: Phe2012 (3.69 Å) and Ile2184 (3.82 Å); no PLIP-defined hydrogen bond.
**QUE**	−7.699	Hydrogen bond: Leu2115 (H···A, 3.37 Å); π–π: Phe2012 (4.08 Å); hydrophobic: Phe1972, Phe2012, Phe2114, Leu2115, Leu2118, Ile2184, and Ile2187 (3.13–3.92 Å).

Supplementary Figure S1: Body weight curves of SD rats during the experiment.

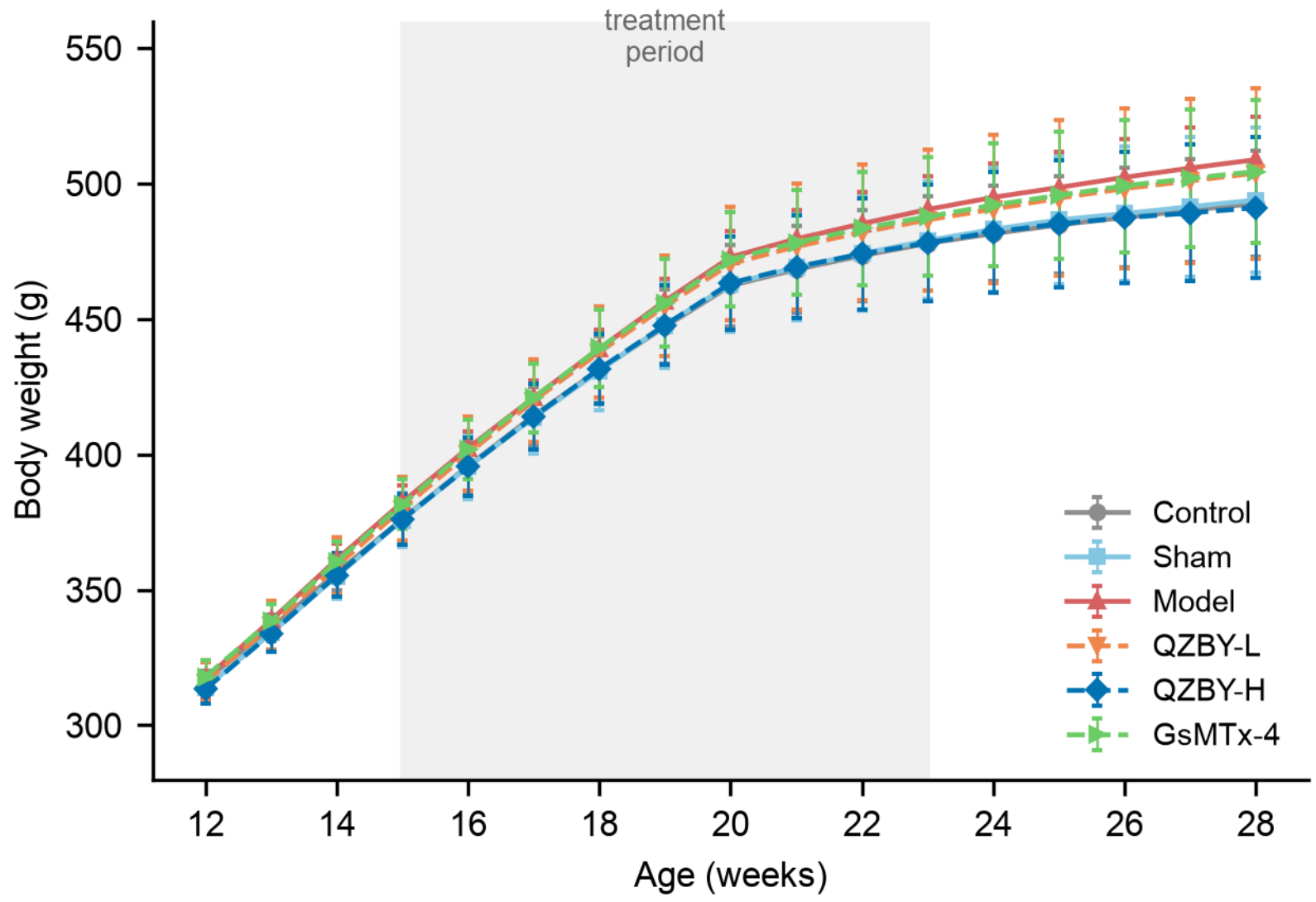

Note: One-way ANOVA at each week, all $P > 0.05$.
